# Noteworthy records of the ticks *Ornithodoros rostratus* and *Amblyomma sculptum* parasitizing *Pteronura brasiliensis* in the central-western region of Brazil, with pathogen investigation notes

**DOI:** 10.1590/S1984-29612024003

**Published:** 2023-12-22

**Authors:** Darci Moraes Barros-Battesti, Marcos Rogério André, Estevam Guilherme Lux Hoppe, Gustavo Seron Sanches, Ricardo Bassini-Silva, Ana Claúdia Calchi, Lívia Oliveira Andrade, Patrícia Parreira Perin, Thiago Fernandes Martins, Ana Carolina Castro-Santiago, Caroline Leuchtenberger, Samara Almeida, Nathalie Foerster, Mariana Furtado, Fernando de Castro Jacinavicius

**Affiliations:** 1 Laboratório de Bioagentes Transmitidos por Vetores – VBBL, Departamento de Patologia, Reprodução e Saúde Única, Faculdade de Ciências Agrárias e Veterinárias, Universidade Estadual Paulista “Júlio de Mesquita Filho” – UNESP, Jaboticabal, SP, Brasil; 2 Departamento de Medicina Veterinária Preventiva e Saúde Animal, Faculdade de Medicina Veterinária e Zootecnia, Universidade de São Paulo – USP, São Paulo, SP, Brasil; 3 Laboratório de Coleções Zoológicas, Instituto Butantan, São Paulo, SP, Brasil; 4 Instituto Pasteur, Secretaria de Estado da Saúde de São Paulo, SP, Brasil; 5 Giant Otter Conservation Fund, Arroio do Meio, RS, Brasil; 6 Instituto Federal Farroupilha, Santa Maria, RS, Brasil.; 7 IUCN Species Survival Commission, Otter Specialist Group, Gland, Switzerland; 8 Instituto Natureza Tocantins, Naturatins, Palmas, TO, Brasil

**Keywords:** Co-parasitism, Argasidae, Ixodidae, giant otter, Pantanal, Co-parasitismo, Argasidae, Ixodidae, ariranha, Pantanal

## Abstract

A male of *Pteronura brasiliensis* (Carnivora: Mustelidae) was found dead on the banks of the Rio Negro, in the Pantanal wetlands of Mato Grosso do Sul state, Aquidauana municipality. Two ticks found attached to its skin were morphologically identified as a second-instar nymph of *Ornithodoros rostratus* (Argasidae) and a male of *Amblyomma sculptum* (Ixodidae). In order to complement the morphological identification, these tick specimens were subjected to DNA extraction, and tested using PCR assays to confirm the molecular identity the specimens. Also, the tick DNA samples were tested and were negative in the PCR assays for all the pathogens tested. We also examined 30 batches, consisting of 174 individuals of *O. rostratus* deposited in the Acari Collection of the Butantan Institute, and we found material from four Brazilian states, including one batch containing 2 males and 2 females from Aquidauana, of Mato Grosso do Sul state, collected from the soil. This was therefore the first record of *O. rostratus* parasitizing *P. brasiliensis* and the first locality record (Aquidauana). Likewise, *A. sculptum* is commonly found in the Pantanal and is reported here for the second time parasitizing the giant otter, which is a host little studied regarding the ectoparasites.

## Resumo

Um macho de *Pteronura brasiliensis* (Carnivora: Mustelidae) foi encontrado morto às margens do Rio Negro, no Pantanal do estado de Mato Grosso do Sul, município de Aquidauana. Dois carrapatos encontrados aderidos à sua pele foram identificados, morfologicamente, como uma ninfa de segundo ínstar de *Ornithodoros rostratus* (Argasidae) e um macho de *Amblyomma sculptum* (Ixodidae). Para complementar a identificação morfológica, esses espécimes de carrapatos foram submetidos à extração de DNA e testados por meio de ensaios de PCR, para confirmar a identidade molecular dos espécimes. Além disso, os extraídos dos carrapatos foram testados e revelaram-se negativos nos ensaios de PCR para todos os patógenos testados. Também foram examinados 30 lotes, compostos por 174 indivíduos de *O. rostratus,* depositados na Coleção Acari do Instituto Butantan, e encontramos material de quatro estados brasileiros, incluindo-se um lote contendo 2 machos e 2 fêmeas de Aquidauana, estado de Mato Grosso do Sul (MS), coletado do solo. Este foi, portanto, o primeiro registro de *O. rostratus* parasitando *P. brasiliensis* e o primeiro registro de localidade (Aquidauana). Da mesma forma, *A. sculptum* é comumente encontrado no Pantanal e relatado aqui, pela segunda vez, parasitando a ariranha, hospedeiro pouco estudado em relação a ectoparasitas.

## Introduction

*Ornithodoros rostratus* Aragão, 1911, is an argasid tick species reported in Brazil, Paraguay, Argentina, and Bolivia. This species belongs to the subgenus *Ornithodoros* (*Pavlovskyella*) Pospelova-Shtrom, 1950, a paraphyletic group of 4 clades and more than 17 species (Mans et al., 2019; Muñoz-Leal et al., 2023).

In addition, *O. rostratus*, along with *Ornithodoros brasiliensis* Aragão, 1923, *Ornithodoros furcosus* Neumann, 1908, and the recently described species *Ornithodoros improvisus* Muñoz-Leal & Venzal, 2023, form the neotropical group of the *Pavlovskyella* subgenus. *Ornithodoros brasiliensis* is endemic to southern Brazil, while *O. furcosus* occurs in the higher-altitude Andean parts of Ecuador, Colombia and Peru, and *O. improvisus* occurs in Chile (Muñoz-Leal et al., 2023). The *Pavlovskyella* species can be found in their hosts' burrows due to their habit of living buried in sand or soft soil near their principal hosts’ habitats. They can also be found living in cellars, stables, and even primitive human dwellings (Clifford et al., 1964). Several *Pavlovskyella* species are vectors of tick-borne relapsing fever caused by *Borrelia* spp. within their geographical ranges (Hoogstraal, 1985).

Until the second half of the twentieth century, *O. rostratus* was abundant in some areas of the Brazilian states of São Paulo, Minas Gerais, Mato Grosso do Sul and Goiás (Aragão, 1936; Aragão & Fonseca, 1961). Since then, this species has not been recorded in any new areas and has only been reported by Cançado et al. (2008), in the locality of Nhecolândia, Mato Grosso do Sul, where it was parasitizing domestic and free-ranging animals, from loose soil on a farm; and by Luz et al. (2019), in the locality of Corumbá, Mato Grosso do Sul, where four nymphs were collected through dragging, and three nymphs and 35 adults from dry‐ice traps. The life cycle of *O. rostratus* was studied by several research as Guglielmone & Hadani (1980), Venzal & Estrada-Peña (2006), Ribeiro et al. (2013) and Costa et al. (2015), and according to these authors, there are five to six nymphal instars. However, the last instar is rare (Ribeiro et al., 2013).

On the other hand, *Amblyomma sculptum* Berlese, 1888, has wide geographical distribution (Martins et al., 2016; de Paula et al., 2022). This species is part of a species complex named “*Amblyomma cajennense* complex”, which includes other five species (Nava et al., 2014). In Brazil, two species of this complex, namely *Amblyomma cajennense* (Fabricius, 1787) (*sensu stricto*) and *A. sculptum*, have been reported.

Even though *O. rostratus* and *A. sculptum* have distinct distribution areas in Brazil, ticks of the *A. cajennense* complex can be found in sympatry in some transition areas. According to Martins et al. (2016), *A. cajennens*e s. s. generally occurs on the border of the Amazon biome and is not found in the thicker parts of the rainforest. *Amblyomma sculptum* is found in the Cerrado, Pantanal and degraded Atlantic Forest biomes, but only one record from the Caatinga biome (Martins et al., 2016) has been reported.

Here, we report the presence of *O. rostratus* and *A. sculptum* in a carcass of a male giant otter (*Pteronura brasiliensis*) for the first and second time, respectively, in the municipality of Aquidauana, state of Mato Grosso do Sul, including pathogen investigation in both species.

## Occurrence Report

During a study carried out by the team responsible for the Giant Otter Conservation Fund, a male of *P. brasiliensis* (Gmelin) (Carnivora: Mustelidae) was found dead on the banks of the Rio Negro at the coordinates 19°34′58″ S and 56°09′44″ W, in the Pantanal region of the state of Mato Grosso do Sul, in the municipality of Aquidauana, in June 2022. Upon inspection, the animal was found to have two specimens of ticks attached to its skin. The ticks were brought to the Vector-Borne Bioagents Laboratory (VBBL), Department of Pathology, Reproduction and One Health, UNESP, Jaboticabal, SP, where they were morphologically identified as a second-instar nymph of *O. rostratus* (Argasidae) (through comparison with specimens from the laboratory’s colony) and a male of *A. sculptum* (Ixodidae).

After morphological identification, both specimens were divided in two pieces, put into a 1.5 µL Eppendorf^®^ microtubes, and following the manufacturer’s protocols, the DNA extraction was performed using the commercial kit - QIAGEN DNeasy Blood & Tissue Kit. Three molecular markers (*18S rRNA*, *16S rRNA* and *cox1*) were targeted for molecular characterization. The following primer pairs were used to target each gene fragment: *18S rRNA* using the primers Mite18S-1F (5’-ATATTGGAGGGCAAGTCTGG-3’) and Mite18S-1R (5’-TGGCATCGTTTATGGTTAG-3’); *16S rRNA* using the primers 16S+1 (5’-CTGCTCAATGATTTTTTAAATTGCTGTGG-3’) and 16S–1 (5’-CCGGTCTGAACTCAGATCAAGT-3’); and *cox1* using the primers LCO1490 (5’-GGTCAACAAATCATAAAGATATTGG-3’) and HCO2198 (5’-TAAACTTCAGGGTGACCAAAAAATCA-3’). PCR reagent concentrations and thermal cycler conditions followed those of the original studies. A negative control (ultrapure water type I; Invitrogen) and a positive control (pool of *Tyrophagus* sp.) were used for each reaction. All positive products were purified with ExoSap-IT (GE Healthcare® Pittsburgh, PA). After extraction, the skin left over from the material was deposited as a voucher for the examined ticks, under the number IBSP 19169A and B - A for *A. sculptum* and B for *O. rostratus*.

Sanger sequencing was performed at the Research Center for the Human Genome and Stem Cells, Institute of Biosciences, University of São Paulo, state of São Paulo. The sequences thus obtained were assembled using the Sequencing Analysis 5.3.1 software and were subjected to BLAST analysis to infer similarities with other tick sequences available in GenBank. Different haplotypes were visually distinguished through alignment using the CLUSTAL W algorithm implemented in Geneious R11.

In addition, molecular tests were performed for the following pathogens, with their respective target genes: order Piroplasmida (*18S rRNA*) (Jefferies et al., 2007); family Anaplasmataceae (*16S rRNA*) (Inokuma et al., 2000); *Bartonella* spp. (*nuoG*) (Colborn et al., 2010); *Borrelia* spp. (FLA) (Stromdahl et al., 2003); *Coxiella* spp. (ITS) (Hoover et al., 1992); *Hepatozoon* spp. (*18S rRNA*) (Spolidorio et al., 2009); *Mycoplasma* spp. (*16S rRNA*) (Maggi et al., 2013); and *Rickettsia* spp. (*gltA*) (Labruna et al., 2004). Negative (Milli-Q water free of DNA) and positive (*Babesia vogeli*-strain Jaboticabal, *Anaplasma phagocytophilum*, *Bartonella henselae*, *Borrelia anserina*, *Coxiella burnetti*, *Hepatozoon procyonis*, *Mycoplasma suis*, *Rickettsia vini* respectively) controls were used for each reaction.

**Figure 1:**
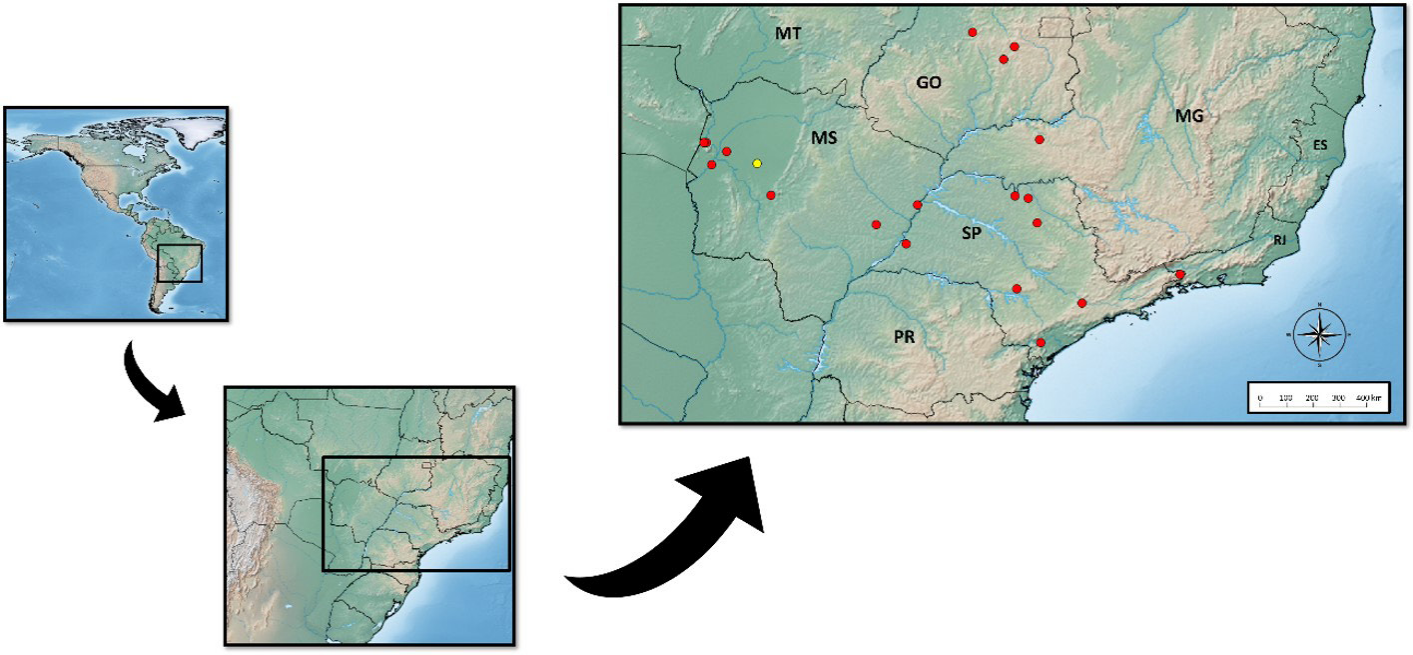
Geographical distribution of *Ornithodoros rostratus* based on records within the IBSP collection. Symbols: red circle – IBSP data (literature records); and yellow circle – new record.

We also examined all specimens of *O. rostratus* deposited in the Acari Collection of the Butantan Institute (IBSP). We found 30 batches of specimens that included 183 individuals (68 females, 47 males, 5 first-instar nymphs, 6 second-instar, 23 third-instar, 28 fourth-instar and 6 fifth-instar), from four Brazilian states: Mato Grosso do Sul – Aquidauana, Corumbá, Ladário, Nhecolândia and Três Lagoas; Goiás – Anápolis and Goiânia; Minas Gerais – Uberlândia; and São Paulo – Araçariguama, Avaré, Bananal, Barretos, Guaraci and Jaboticabal. The geographical distribution of this species based on material deposited in the IBSP collection and the new locality record is shown in Figure 1.

**Figure 2:**
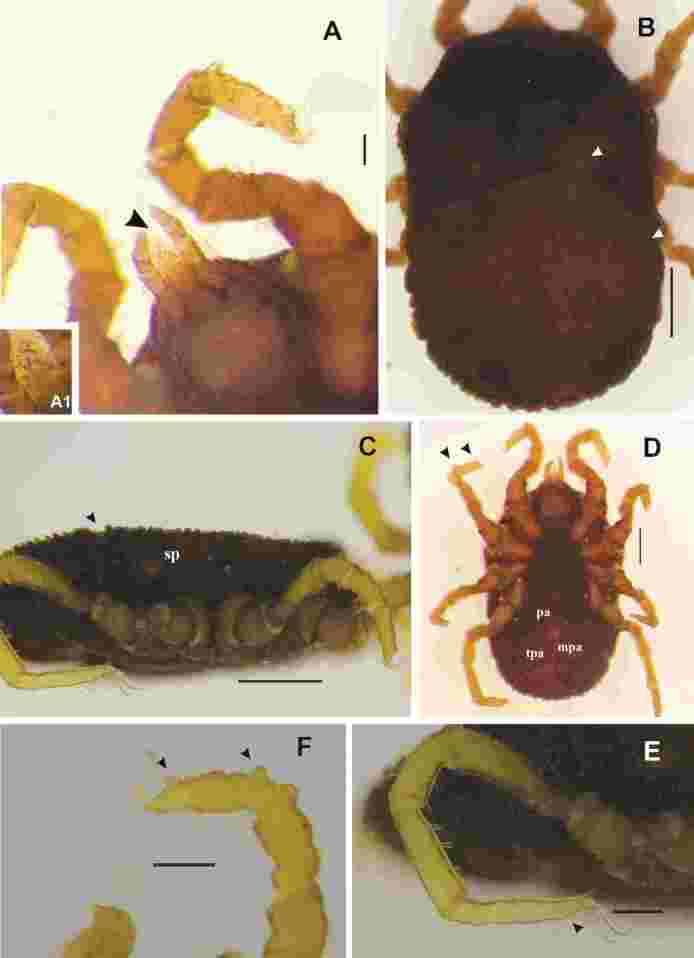
*Ornithodoros rostratus*, second nymphal instar (N2). A: Gnathosoma, black arrow highlights the hypostome, bar 100 µm. A1: Hypostome with two rows of strong teeth. B: Idiosoma, dorsal view, white arrow highlights the dorsoventral groove and oval discs and mammillae, bar 500 µm. C: Idiosoma, lateral view, black arrow highlights the dorsoventral groove, bar 500 µm. D: Idiosoma, ventral view, black arrows highlight two strong dorsal humps on tarsus II, bar 500 µm. E: Tarsus I, black arrows highlight two strong dorsal humps, bar 200 µm. F: Tarsus IV, black arrow highlights the distal hump, bar 200 µm. Abbreviations: mpa, median postanal groove; pa, preanal groove; sp, spiracular plate; tpa, transverse postanal groove.

The nymph (N2) was identified by comparisons with nymphs of all instars (six instars) obtained from the colony of *O. rostratus*. Detailed descriptions of each nymphal instar of this species are in preparation. The colony of *O. rostratus* used for identification of nymphs started in 2010, from specimens collected in 2006 to 2007, in area of the Brazilian Pantanal where is located the EMBRAPA’s farm, Nhecolândia municipality, Mato Grosso do Sul state (18°59′S and 56°39′W). The free-living ticks were trapped by CO_2_ traps according to Cançado et al. (2008), and after that these alive ticks were donated by PHD Cançado to the Laboratory of Parasitology in the Instituto Butantan, São Paulo State, to start a colony in 2010. Since that, the colony of *O. rostratus* has been maintained in vials and packed in a biological oxygen demand (BOD) incubator at 27 ± 1º C and 90 ± 10% humidity, in the same laboratory. Also, this N2 nymph (Figure 2) had the following characteristics: idiosoma outline with oval discs and mammillae; length from anterior to posterior body margin 2679.28 mm, breadth 1537.20; legs with dorsal humps on tarsi; two strong and protuberant dorsal humps on tarsus I, one proximal (close to the tibia) and one distal (close to Haller’s organ); also strong and protuberant humps on tarsi II and III; tarsus VI with very small, almost imperceptible, distal and proximal dorsal humps; all coxae with smooth surface, upper and lower borders surrounded by small mammillae that were present on supracoxal folds; preanal, transverse postanal, median postanal and dorsoventral grooves present; spiracular plate rounded; gnathosoma, including palpi and hood, visible dorsally; hypostome blunt apically, dentition 2/2.

Furthermore, we confirmed the two species molecularly. For *O. rostratus*, we were able to obtain the three markers mentioned above, whereas for *A. sculptum*, we only obtain the *16S rRNA* gene. The GenBank accession number and the respective genes are as follows: for *O. rostratus* - OR484738 (18S rRNA), OR484739 (16S rRNA) and OR484740 (*cox1*); and for *A. sculptum* - OR484742 (16S rRNA). No selected pathogen DNA was detected in the tick specimens.

The obtained partial sequence of the *O. rostratus* N2 for the gene 16S rRNA was 100% identical to *Ornithodoros rostratus* (JN887882), while the partial sequence of the 18S rRNA was 99.80% identical to *Ornithodoros rostratus* (KC769605), and the partial sequence of the *cox1* was 99.71% identical to *Ornithodoros rostratus* (NC_023372). The 16S rRNA partial sequence of the *A. sculptum* was 99.76% identical to *Amblyomma sculptum* (MW219724).

Giant otters are semiaquatic and social animals. They display allogrooming not only to reinforce their social bonding, but also for skin cleaning (Duplaix, 1980). Groups of giant otters dig dens along the banks of water bodies (Leuchtenberger et al., 2015), and these are also visited by other vertebrates (Leuchtenberger, pers. comm.). Thus, giant otters’ dens can form an environment of tick infestation and pathogen transmission, despite these animals’ allogrooming.

Although *O. rostratus* was collected in abundance in the past, it has practically disappeared in many areas, such as the state of São Paulo. However, our finding of a nymph on a giant otter in the Pantanal suggests that this argasid species is still present in central Brazil, mainly in areas where domestic animals coexist with wild fauna. Likewise, *A. sculptum* is commonly found in the Pantanal and is reported here for the second time parasitizing the giant otter, which is a host little studied with regard to ectoparasites. Recently, in the same area, three specimens of *A. sculptum* were collected from a carcass of a giant otter that had been found dead, like in the present study (Soresini et al., 2023). In addition to *A. sculptum*, a nymph of *A. cajennense* s. s. was found parasitizing an individual of *P. brasiliensis* in the Brazilian Amazon region (Rosas et al., 2016). Although different primers were used for pathogen DNA screening, both specimens were negative for the selected vector-borne agents.
