# ERRATUM: Noteworthy records of the ticks *Ornithodoros rostratus* and *Amblyomma sculptum* parasitizing *Pteronura brasiliensis* in the central-western region of Brazil, with pathogen investigation notes

**DOI:** 10.1590/S1984-29612024009

**Published:** 2024-02-12

In the article "Noteworthy records of the ticks *Ornithodoros rostratus* and *Amblyomma sculptum* parasitizing *Pteronura brasiliensis* in the central-western region of Brazil, with pathogen investigation notes", DOI https://doi.org/10.1590/S1984-29612024003, published in issue 1, volume 33, 2024, the Brazilian Journal of Veterinary Parasitology, e014523, which reads:

Gustavo Seron

Read up:

Gustavo Seron Sanches

